# Assessment of clinical radiosensitivity in patients with head-neck squamous cell carcinoma from pre-treatment quantitative ultrasound radiomics

**DOI:** 10.1038/s41598-021-85221-6

**Published:** 2021-03-17

**Authors:** Laurentius Oscar Osapoetra, Archya Dasgupta, Daniel DiCenzo, Kashuf Fatima, Karina Quiaoit, Murtuza Saifuddin, Irene Karam, Ian Poon, Zain Husain, William T. Tran, Lakshmanan Sannachi, Gregory J. Czarnota

**Affiliations:** 1 Department of Radiation Oncology, Sunnybrook Health Sciences Centre, T2 167, 2075 Bayview Avenue, Toronto, ON M4N3M5 Canada; 2 Department of Radiation Oncology, University of Toronto, Toronto, Canada; 3 Physical Sciences, Sunnybrook Research Institute, Toronto, Canada; 4 Evaluative Clinical Sciences, Sunnybrook Research Institute, Toronto, Canada; 5 Department of Medical Biophysics, University of Toronto, Toronto, Canada

**Keywords:** Cancer, Computational biology and bioinformatics, Oncology

## Abstract

To investigate the role of quantitative ultrasound (QUS) radiomics to predict treatment response in patients with head and neck squamous cell carcinoma (HNSCC) treated with radical radiotherapy (RT). Five spectral parameters, 20 texture, and 80 texture-derivative features were extracted from the index lymph node before treatment. Response was assessed initially at 3 months with complete responders labelled as early responders (ER). Patients with residual disease were followed to classify them as either late responders (LR) or patients with persistent/progressive disease (PD). Machine learning classifiers with leave-one-out cross-validation was used for the development of a binary response-prediction radiomics model. A total of 59 patients were included in the study (22 ER, 29 LR, and 8 PD). A support vector machine (SVM) classifier led to the best performance with accuracy and area under curve (AUC) of 92% and 0.91, responsively to define the response at 3 months (ER vs. LR/PD). The 2-year recurrence-free survival for predicted-ER, LR, PD using an SVM-model was 91%, 78%, and 27%, respectively (p < 0.01). Pretreatment QUS-radiomics using texture derivatives in HNSCC can predict the response to RT with an accuracy of more than 90% with a strong influence on the survival.

**Clinical trial registration:** clinicaltrials.gov.in identifier NCT03908684.

## Introduction

Radiomics is an emerging field in oncology involving advanced computational imaging analysis and typically involves the application of artificial intelligence for meaningful interpretation of data^1^. Tumour images are now recognized as more than greyscale images, and computer vision can unfold information that can be linked with underlying genotypic and phenotypic features^2^. Imaging forms an integral role in oncology in diagnosis, disease staging, treatment planning, the assessment of treatment response, and tumour surveillance. Standard imaging modalities involve morphological-based techniques like ultrasonography (US), computed tomography (CT), magnetic resonance imaging (MRI) or functional imaging like positron emission tomography (PET) or functional MRI (fMRI). The use of radiomics to serve as potential noninvasive biomarkers in risk stratification, in prediction and monitoring of treatment response has generated interest to develop strategies towards precision oncology^1,3^.

Head and neck malignancies accounted for 890,000 new cases worldwide (seventh most common globally) and 450,000 deaths in 2018^4^. Radical radiotherapy (RT) with or without concurrent chemotherapy forms the primary treatment for a majority of patients with head and neck squamous cell carcinomas (HNSCC) arising from the oropharynx, hypopharynx, or larynx leading to excellent organ preservation^5^. Locally advanced cancer is present in approximately 40–60% of patients during presentation, which involves advanced primary disease and/or regional lymph nodes (LN)^5,6^. In the past decades, technological advances in RT with intensity-modulated radiotherapy (IMRT) and image-guided radiotherapy (IGRT) have led to a reduction of radiation toxicities like xerostomia^7^. The survival outcome of patients with locally advanced HNSCC remains compromised despite such advancements. As a result, there is increasing interest regarding the use of response-guided adaptive RT, with several ongoing trials investigating dose-escalation strategies for patients deemed to be non-responders^8,9^.

As imaging has a crucial role in the management of HNSCC, radiomic analysis has been undertaken for different imaging modalities like PET, CT, and MRI^10–12^. Study endpoints have been variable with some studies investigating molecular characteristics, while others have linked radiomic features with survival outcomes with encouraging results. Quantitative ultrasound (QUS) uses data typically not interpreted by clinical B-mode US devices. QUS uses raw radiofrequency (RF) data, which can provide information related to tissue microstructure elastic properties and underlying biology^13^. Tumour treatment response can be detected using QUS much earlier than conventional imaging due to ongoing changes in elastic properties associated with cell death^14,15^. Radiomic analysis of QUS has proven to be useful in the determination of response to neoadjuvant chemotherapy in breast cancer^16–19^.

In previous studies, QUS obtained before and during treatment was shown to be promising in predicting treatment response for head-neck malignancies^20,21^. In a cohort of 32 patients, pre-treatment QUS could predict the response at 3 months with an accuracy of 88%^20^. In a subsequent study including 36 patients, the classifier performance was improved when QUS features were obtained during radiotherapy as early as after 1 week of treatment compared to pr-treatment^21^. In the current study, the number of patients has been increased to 59, and includes a homogeneous group of HNSCC (excluding other tumour histologies included in the previous study such as nasopharynx, parotid, and others). Also, the study methodology has significant development incorporating third-order imaging features (texture derivatives), using more advanced machine learning classifiers, and final correlation with clinical outcomes. In addition, the endpoint of final response beyond 3 months in partial responders was analyzed in the present study. The imaging features used in the study were spectral parameters, texture of spectral parameters (QUS-Tex^1^), and second-order texture analysis of QUS-Tex^1^ features (QUS-Tex^1^-Tex^2^). Five spectral parameters were used-mid-band fit (MBF), spectral slope (SS), spectral intercept (SI), average scatterer diameter (ASD), and average acoustic concentration (AAC). The machine learning classifiers included Fischer’s linear discriminant analysis (FLD), *k-*nearest neighbours (KNN), and support vector machines-radial basis function (SVM-RBF). Four textural features of contrast (CON), correlation (COR), energy (ENE), and homogeneity (HOM) were analyzed. To the best of our knowledge, this is the first study of QUS-radiomics using higher-order imaging features to predict different groups of radiation responses, which were linked to clinical outcomes.

## Results

### Clinical characteristics

A total of 59 patients with node-positive HNSCC were included in the current analysis. The different patient, disease, and treatment-related features have been summarized in Table 1. None of the clinical features were significantly distributed between the three response groups (ER/LR/PD). In the entire group, the median age was 61 years (range, 39 to 80 years), with 12 patients being 70 years or above. The median size of the lymph nodes was 3.1 cm (range 1.3 to 7 cm), without any significant difference between the three response groups. The most common primary site was oropharynx in 42, followed by carcinoma unknown primary in 8, larynx in 6, and hypopharynx in 3 patients. The human papillomavirus (HPV) p16 immunostaining was available in 44 patients, with positive staining in 38 (86%). Concurrent chemotherapy was used in 49, cetuximab (without chemotherapy) in 2, and RT alone in 8 patients. All patients included in the current study completed the scheduled course of RT.

**Table 1:** Clinical characteristics and survival outcomes for the three response groups.

Parameter	Early responder (n = 22)	Late responder (n = 29)	Persistent/progressive disease (n = 8)	p-value
Radiation response category	Highly radiosensitive	Intermediate radiosensitivity	Radioresistant	
Age median (range)	66 (47–80) years	59 (39–79) years	62 (57–78) years	0.57
**Gender**				
Male	21	27	8	
Female	1	2	0	0.73
**ECOG performance status**				
0	6	9	2	0.93
1	16	20	6	
**Primary site**				
Oropharynx	15	22	5	
Larynx	3	1	2	0.17
Hypopharynx	2	0	1	
CUP	2	6	0	
**HPV status**				
Positive	18	17	3	
Negative	1	4	1	0.18
Unknown	3	8	4	
**T-stage**				
T0	2	6	0	
T1	8	5	0	
T2	4	8	4	0.28
T3	2	4	1	
T4	6	6	3	
**N-stage**				
N1	11	11	2	
N2	10	13	2	0.07
N3	1	5	4	
**Number of nodes**				
Median (range)	1 (1–3)	1 (1–5)	1 (1–4)	0.23
**Concurrent therapy**				
Cisplatinum	17	23	5	
Carboplatinum	1	2	0	
Cisplatinum > carboplatinum	0	1	0	0.66
Cetuximab	1	0	1	
None	3	3	2	
**2-year RFS**	96%	74%	13%	< 0.01
**2-year OS**	100%	88%	45%	< 0.01

*ECOG* Eastern cooperative oncology group, *CUP* carcinoma unknown primary, *HPV* human papillomavirus, *RFS* recurrence-free survival, *OS* overall survival.

### Clinical outcomes

During the first response evaluation at 3 months, 22 patients had a complete response and were designated as early responder (ER), as described earlier. For the remaining 37 patients, 29 were designated as late responder (LR) as they had complete disease resolution with a median of 6 months from RT completion (range 4 to 10 months), whereas 8 patients had persistent/progressive disease (PD) involving treated target disease (primary and nodal target volumes). Median follow up for all the patients was 32 months (range 5 to 64 months). At follow up for the current study, 18 patients had recurrent disease (local-1, nodal-5, distant-9, local-nodal-1, local-nodal-distant-2). The 2-year and 5-year recurrence-free survival (RFS) for all patients was 72% and 68%, respectively. The 2-year and 5-year overall survival (OS) for the entire cohort was 86% and 61%, respectively. Both the RFS and OS were significantly different between the three response groups (Table 1). The 2-year RFS for the ER, LR, and PD was 96%, 74%, and 13%, respectively (p < 0.01).

### Feature analysis

**Figure 1:**
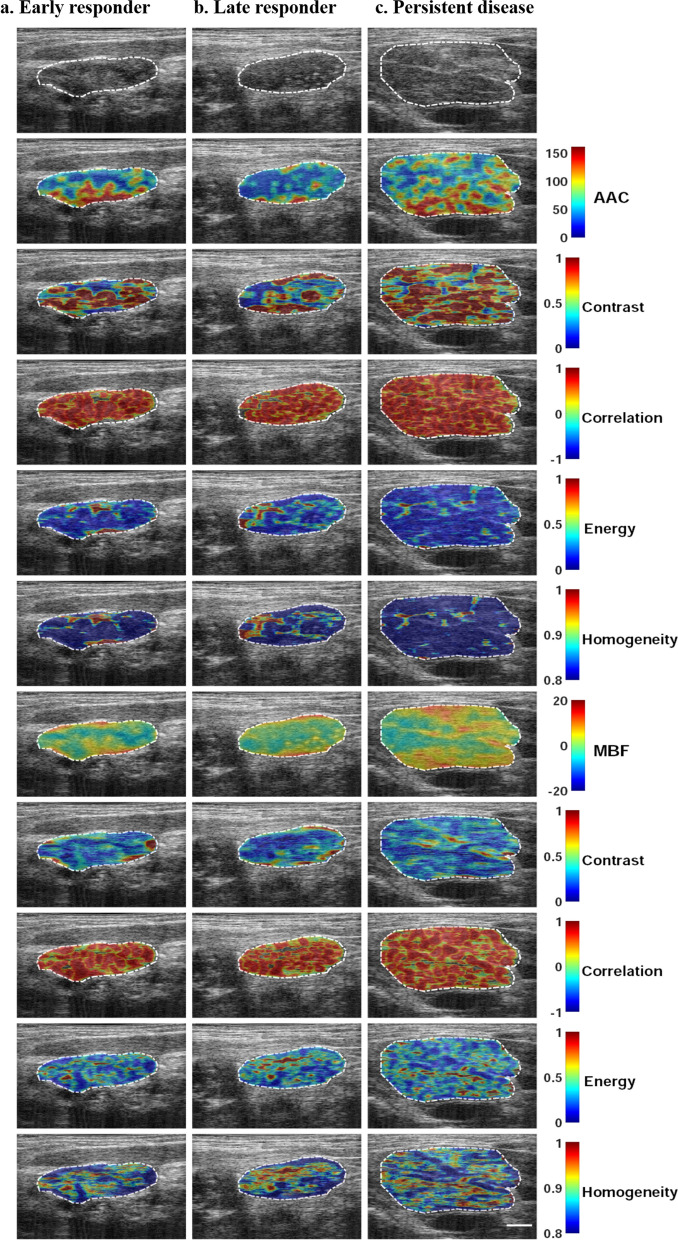
Representative ultrasound B-mode (uppermost row), QUS spectral parametric maps of AAC and MBF, and the corresponding texture images from one patient in each of the three response groups-early responder (**a**), late responder (**b**), and persistent disease (**c**). QUS parametric images include the largest involved lymph node (central region bounded by closed dotted white curve). The colour bar ranges are 0 to 150 dB/cm^3^ for AAC, − 20 to 20 dB for MBF and arbitrary unit for the texture features. The scale bar represents 1 cm.

Representative B-mode images and the corresponding representative QUS and QUS-texture maps are presented in Fig. 1 for three patients, one each from the ER, LR, and PD groups. Obvious intratumoral heterogeneity was evident from the spectral parametric maps and their texture maps. Figure 1 demonstrates typical hypoechogenicity for the tumour and apparent heterogeneity in quantitative ultrasound parameters.

Table 2 indicates the features that exhibited statistically significant differences between the different response groups. Three months after RT completion, 12 features were found to be significantly different between the complete response (ER) and partial/non-responder (LR/PD) groups. These included two spectral parameters (MBF, SI), one QUS-Tex^1^ feature (MBF-CON), and 9 QUS-Tex^1^-Tex^2^ features. Between the LR and PD groups, a different set of 12 features had different distributions. One spectral feature (ASD), three QUS-Tex^1^ features (AAC-CON, ASD-CON, and AAC-HOM), and 8 QUS-Tex^1^-Tex^2^ features were found to have different values between these two response groups. The scatter plots of all the 105 features between the binary response groups have been shown in Supplementary Fig. S1.

**Table 2:** Imaging biomarkers that demonstrate statistically significant differences (p < 0.05) between the response groups at different time-points.

Features	Group 1 (mean ± SEM)	Group 2 (mean ± SEM)	p-value
	Complete responder	Partial/non-responder	
**Response at 3 months** **Complete responder (early responder) (n = 22) vs partial/non-responder (n = 37)**			
MBF-COR-COR	0.67 ± 0.01	0.64 ± 0.01	0.001
MBF-HOM-CON	1.29 ± 0.03	1.12 ± 0.04	0.004
SI-COR-COR	0.63 ± 0.01	0.61 ± 0.00	0.006
SI-HOM-CON	1.41 ± 0.03	1.26 ± 0.04	0.011
SS-COR-COR	0.62 ± 0.00	0.60 ± 0.00	0.017
SS-HOM-CON	1.41 ± 0.04	1.26 ± 0.04	0.018
MBF	0.97 ± 1.98	− 2.92 ± 1.40	0.027
MBF-CON	0.59 ± 0.03	0.52 ± 0.03	0.031
SI	15.20 ± 1.90	10.65 ± 1.13	0.032
MBF-CON-COR	0.70 ± 0.02	0.66 ± 0.01	0.035
ASD-COR-COR	0.62 ± 0.00	0.61 ± 0.00	0.042
ASD-HOM-CON	1.40 ± 0.03	1.32 ± 0.04	0.046

	LR (mean ± SEM)	PD (mean ± SEM)	p-value
**Final response** **Late responder (n = 29) vs persistent/progressive disease (n = 8)**			
AAC-COR-ENE	0.18 ± 0.00	0.21 ± 0.01	0.000
AAC-ENE-HOM	0.62 ± 0.01	0.67 ± 0.01	0.001
AAC-CON	1.08 ± 0.10	1.91 ± 0.33	0.002
AAC-ENE-CON	7.76 ± 0.34	5.15 ± 0.57	0.002
AAC-COR-HOM	0.54 ± 0.00	0.56 ± 0.01	0.003
AAC-ENE-ENE	0.24 ± 0.01	0.32 ± 0.03	0.006
SS-HOM-ENE	0.32 ± 0.00	0.30 ± 0.01	0.008
SI-HOM-HOM	0.73 ± 0.00	0.72 ± 0.00	0.009
ASD-CON	1.08 ± 0.08	1.62 ± 0.25	0.009
AAC-HOM	0.79 ± 0.01	0.71 ± 0.04	0.013
ASD	95.49 ± 6.23	67.34 ± 9.40	0.014
SS-HOM-HOM	0.73 ± 0.00	0.72 ± 0.00	0.015

*SEM* standard error mean, *MBF* midband fit, *SI* Spectral intercept, *SS* spectral slope, *ASD* average scatterer diameter, *AAC* average acoustic concentration, *COR* correlation, *CON* contrast, *HOM* homogeneity, *ENE* energy.

### Classification performances

**Figure 2:**
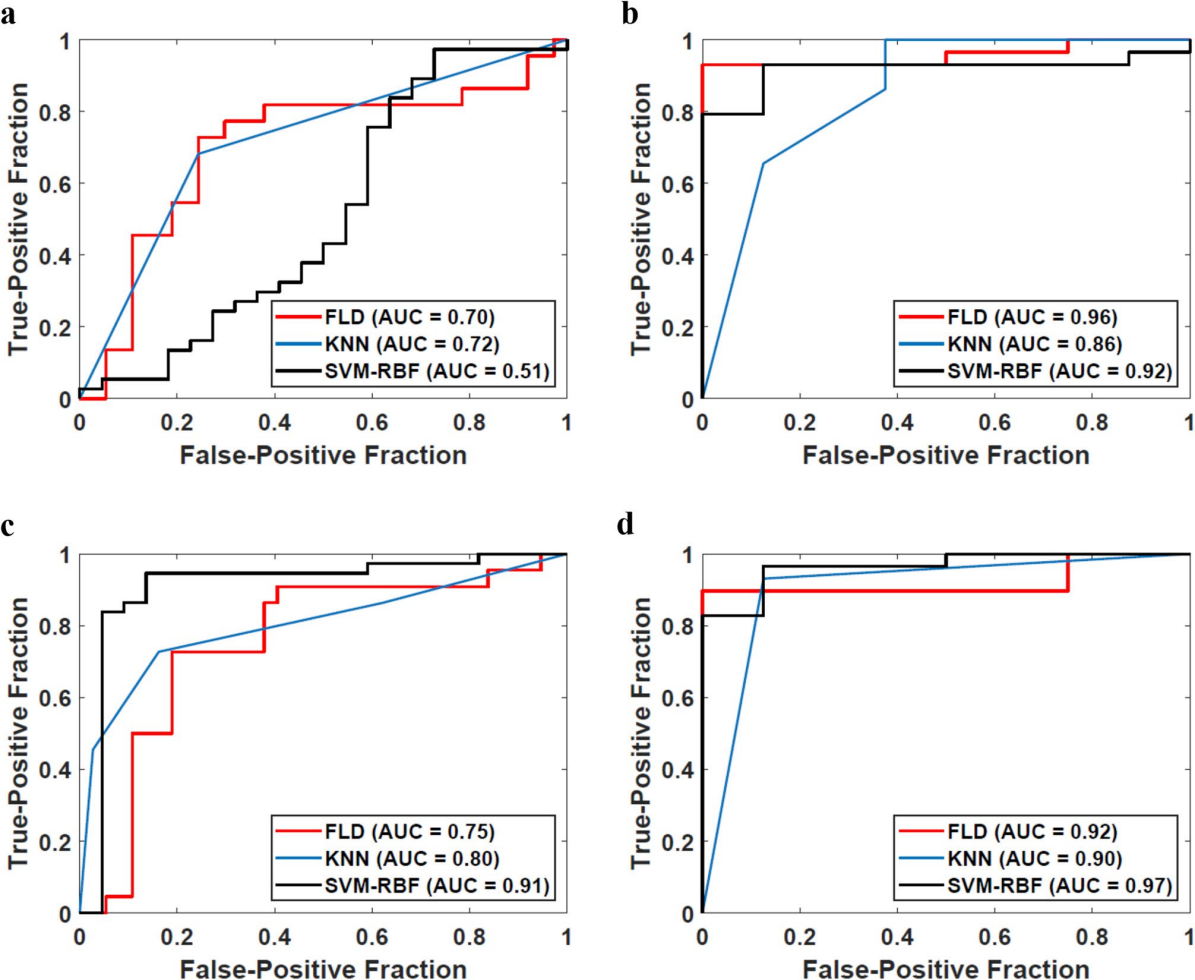
ROC plots of predictive models for different endpoints developed using spectral and texture features alone (upper row) and those developed using all the spectral, texture, and texture-derivate features (lower row). The endpoints considered are 3-month complete responder versus partial responder/non-responder (**a**,**c**), late responder versus persistent/progressive disease (**b**,**d**). Three standard classification algorithms that include FLD, KNN, and SVM-RBF were evaluated as indicated in the inset legend. The classification models that include texture-derivate features (lower row) achieved higher AUC values in general compared to those developed without texture-derivate features.

**Figure 3:**
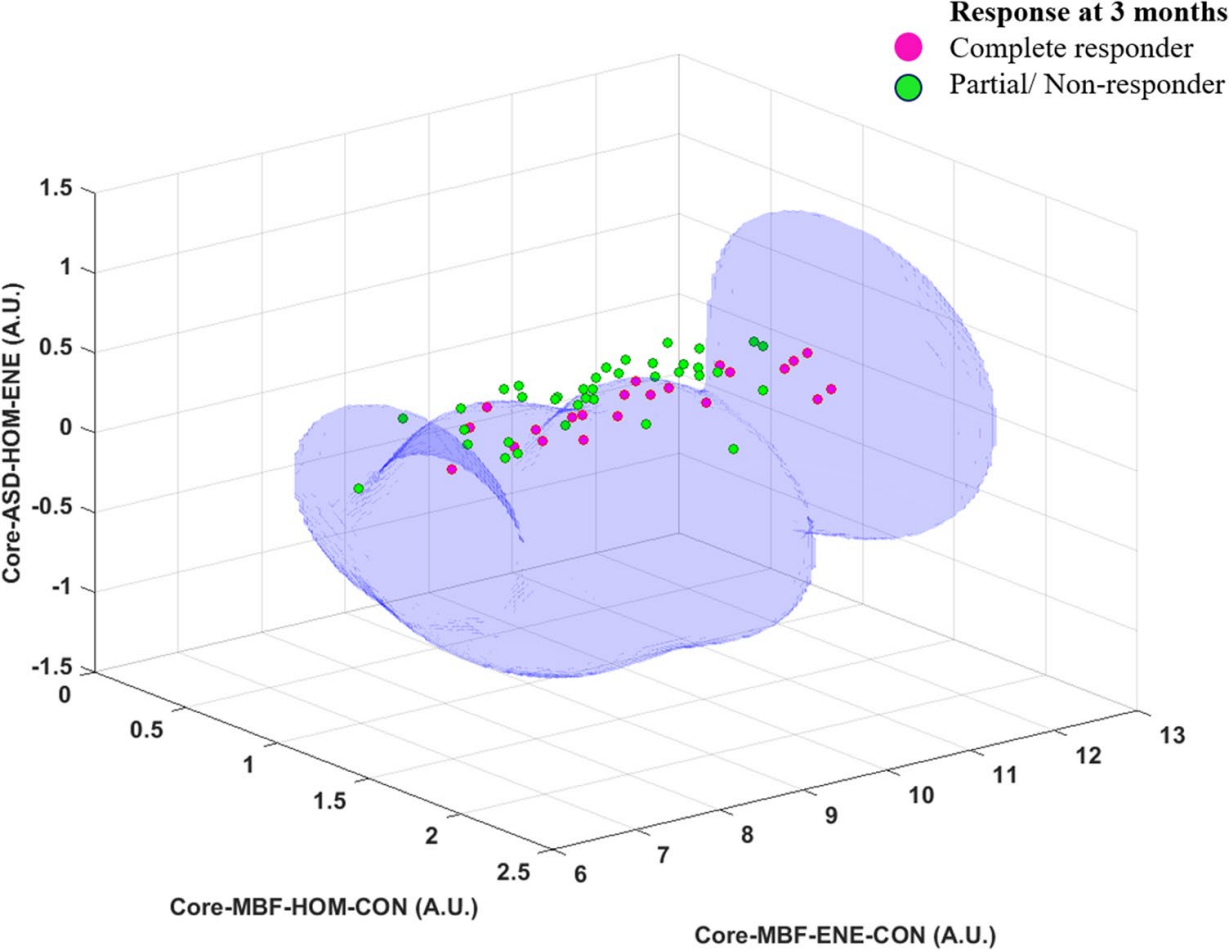
Hyperplane plot with the decision boundary based on support vector machine classifier using three features to differentiate the complete responders from partial/non-responders at three months following radiotherapy completion.

Table 3 presents the classifier performance using the three classifiers for the two binary response groups. The SVM-RBF model demonstrated the best results in differentiating the ER from the LR/PD group with a sensitivity, specificity, accuracy, and AUC of 86%, 95%, 92%, and 0.91, respectively. The three features selected for building the classifier model were QUS-Tex^1^-Tex^2^ parameters (MBF-HOM-CON, MBF-ENE-CON, ASD-HOM-ENE). For the SVM model, the accuracy was improved by 3% with parameter tuning. To study the influence of texture-derivatives on the classifier performance, the analyses were carried out in a step-wise manner, once using spectral and QUS-Tex^1^ features only and then finally, with all the 105 features incorporating QUS-Tex^1^-Tex^2^ features. The inclusion of texture-derivatives led to a significant improvement of the classifier performances. Without the texture-derivatives, the AUC was 0.70, 0.72, 0.51 for FLD, KNN, and SVM-RBF, which was improved to 0.75, 0.80, and 0.91 when all the QUS-Tex^1^-Tex^2^ features were included. The ROC plots using the three classifiers for the two different endpoints (with and without texture-derivatives) are shown in Fig. 2. The pictorial representation of the hyperplane plot using the SVM-RBF model to classify the two response groups at 3 months has been presented in Fig. 3.

**Table 3:** Performance of various machine learning classifiers with the best features selected for the different response groups.

Classifier	Sensitivity (%)	Specificity (%)	Accuracy (%)	AUC	Features selected
**Response at 3 months** **Complete responder (early responder) (n = 22) vs partial/non-responder (n = 37)**					
FLD	73	81	78	0.75	MBF-HOM-CON MBF SI-CON-ENE
KNN	73	84	80	0.80	SS-COR-COR MBF-ENE-HOM NA
SVM-RBF	86	95	92	0.91	MBF-HOM-CON MBF-ENE-CON ASD-HOM-ENE
**Final response** **Late responder (n = 29) vs persistent/progressive disease(n = 8)**					
FLD	86	100	89	0.92	AAC-ENE-HOM AAC-HOM-CON
KNN	93	88	92	0.90	AAC-HOM ASD-ENE-HOM
SVM-RBF	97	88	95	0.97	SS SS-HOM-CON

*FLD* Fischer’s linear discriminant analysis, *KN k*-nearest neighbour, *SVM-RBF* support vector machine-radial based function, *MBF* midband fit, *SI* spectral intercept, *SS* spectral slope, *ASD* average scatterer diameter, *AAC* average acoustic concentration, *COR* correlation, *CON* contrast, *HOM* homogeneity, *ENE* energy.

In order to differentiate the LR from PD, the SVM-RBF performed better than the other two models with sensitivity, specificity, accuracy, and AUC of 97%, 88%, 95%, and 0.97, respectively. The two selected features in the model were SS and SS-HOM-CON. Similar to the previous endpoint, the inclusion of texture derivatives improved the AUC for the SVM-RBF model from 0.92 to 0.97.

**Figure 4:**
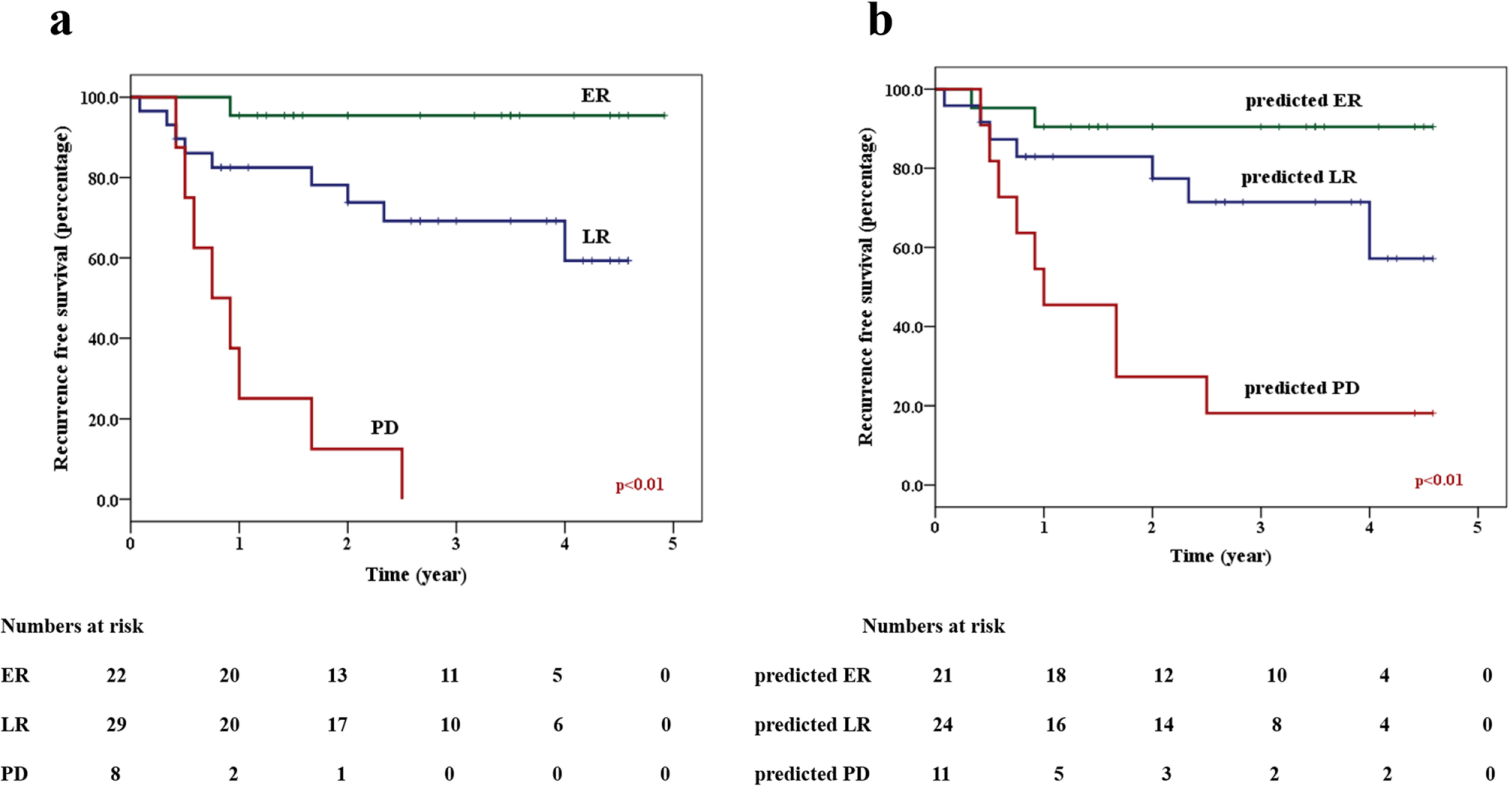
Kaplan Meier survival plots showing the recurrence-free survival for the three predicted groups using a support vector machine radiomics model.

Finally, each patient was assigned to one of the three predicted response groups using the SVM-RBF model. The 2-year RFS for the predicted ER, predicted LR, and predicted PD groups were 91%, 78%, and 27%, respectively (p < 0.01). Figure 4 shows the comparison of the RFS survival plots generated using the actual response groups and the radiomics-predicted response groups.

## Discussion

Head and neck malignancies represent a diverse group of malignancies arising commonly from the epithelial lining of the upper aerodigestive tracts and associated glandular structures^5^. Outcomes in HNSCC are strongly influenced by clinical-pathological factors like the site of primary disease, disease stage, performance status of the patient, and causative factors like tobacco abuse or HPV. For patients with locally-advanced HNSCC or node-positive disease, the prognosis is poor, with an estimated 5-year survival rate being approximately 50% or less^5^. Also, with the presence of vital anatomical and physiological structures in the head and neck region, in patients cured of their disease, long-term treatment-related late toxicities can have significant adverse effects on quality of life^22,23^. There is an unmet need for the development of reliable biomarkers, which can help in better risk-stratification so that existing treatment strategies can be tailored accordingly. The introduction of computational techniques and artificial intelligence in imaging and medicine has led to the development of the field of radiomics, with standard imaging modalities producing new potential noninvasive biomarkers. In the current study, the role of ultrasound before radiation treatment was investigated in predicting final clinical response to therapy, using higher-order imaging features.

In the past few years, there had been an increasing number of studies investigating the role of radiomics in HNSCC, primarily involving CT, PET, or MRI^10–12^. The majority of the radiomic studies have investigated endpoints like molecular characteristics (HPV status) and clinical outcomes like local control, distant metastasis, and overall survival. Although favourable results have been obtained from most of the studies, there has been variance with regards to image processing, feature selection strategies, machine learning algorithms, and validation approaches^24^. A recent multi-institutional has indicated the utility of pre-operative contrast-enhanced CT-based radiomics in predicting histopathological extranodal extension^25^. In a study involving 128 patients with head and neck malignancies, Bogowicz et al*.* had shown radiomics analysis of CT features from the primary tumour and lymph nodes to correlate with loco-regional control^26^. Similarly, in a study involving 300 patients with HPV-related oropharyngeal cancers, Kwan et al*.* associated distant metastasis with radiomic features obtained from radiation planning CT images^27^. Vallières et al. had demonstrated that radiomic analysis of pretreatment PET and CT images could be used in a risk assessment for loco-regional recurrence and distant metastasis in a study of 300 patients with head neck malignancies undergoing radical intent RT^28^. Fewer imaging studies have investigated response to therapy as a study endpoint in head neck malignancies. Liu et al*.* performed texture analysis of T1, T2, and diffusion MR sequences in 53 patients with NPC treated with chemoradiation, which was able to predict the treatment response with an accuracy of approximately 90%^29^. Similarly, Wang et al*.* used pretreatment MRI in 120 patients with NPC to predict response to induction chemotherapy^30^.

Ultrasonography is frequently used in the management of HNSCC, more commonly indicated in the characterization of suspicious neck nodes, as well as to guide biopsy or cytology procedures for histopathological confirmation^31,32^. B-mode US is more popularly used in clinical scenarios. The use of QUS involves technically identical ultrasound devices, but with raw RF data collected and analyzed quantitatively. QUS detects the elastic properties of the tissue microstructure and is influenced by parameters like cell size, shape, and organization^33^. Microcellular elastic properties have been demonstrated to be different across different grades of tumours, which have distinct biological behaviour^34^. In tissue characterization, QUS techniques have been proven to be extremely sensitive in detecting ongoing changes with treatment-induced cell death since it leads to changes in nuclear and cell structures properties with cell fragmentation, pyknosis, and the formation of apoptotic bodies, ultimately changing scatterer elastic properties^14,15^. Clinical studies investigating QUS-radiomics indicated this methodology to be useful in predicting response to neoadjuvant chemotherapy (NAC) in patients with locally advanced breast cancer (LABC) before starting treatment^16^. Similarly, response changes with NAC can be detected as early during the first week of treatment, correlating with the final response obtained from histopathological examination months after the treatment is started^17^. This methodology is being evaluated in a randomized clinical trial of QUS-radiomics guided adaptive chemotherapy, where patients predicted to have inadequate response can have modifications made to their NAC (clinicaltrials.gov identifier NCT04050228). The application of QUS imaging for head and neck malignancies is a relatively new technique, with initial results from 32 patients showing a predictive accuracy of 88% using spectral and texture features (18). Compared to other imaging modalities, QUS-based radiomics has the advantage of rapid scan acquisition that can be performed with a portable device, with excellent patient compliance. A QUS-based approach is expected to provide more biologically-linked information compared to other imaging like CT or MRI as the imaging information can be tuned to pick up details from a smaller foci of cells and is sensitive to approximately 10–20 cell diameters using a mean frequency of 6 MHz) (24).

In the current study, using an SVM-based model, the accuracy of classification between complete responders versus non-responders/partial responders at 3 months and late responders vs persistent/progressive disease patients was 92% and 95%, respectively. There were 12 features at each time having a differential distribution between the two binary groups. At the 3 month time, most of the features that were different between the two response groups were related to the MBF parameter. Mid-band fit denotes the value of linear fit at the central frequency and is influenced by scatterer size, shape, and organization (24). The 3-feature set used in the classification model used texture-derivatives of one ASD parameter and 2 MBF parameters. ASD is dependant on cell size, suggesting a differential structural organization between the two response groups, which impacted radiation response. Between the LR and PD response groups, the values were seen to be different for many of the AAC parameters, which accounts for the density of scatterers, their organization, and their elastic properties. The 2-feature SVM-model used SS and SS-texture-derivative features, with SS representing information related to cell shape and size. It is essential to note the majority of features selected in model development were texture-derivatives suggesting intratumoral heterogeneity to have an important implication on response to radiation and clinical outcomes. Also, the inclusion of higher-order texture features led to an improvement in classifier performance, indicating a better representation of spatial heterogeneity with third-order imaging analysis.

Response to radiation had a strong influence on clinical outcomes (both RFS and OS). The patients with an early complete response within the first 3 months are likely to harbour disease sensitive to RT and had the best outcomes. Patients who exhibited a delayed response demonstrated an intermediate prognosis. As expected, patients with residual or progressive disease in an irradiated volume indicated radioresistant disease and showed the poorest RFS and OS amongst the patient groups. The radiomics model developed here can be utilized as a tool for predicting clinical radiosensitivity, with links to ultimate survival outcomes. The development of treatment-escalation strategies has been explored elsewhere using response-guided adaptive radiotherapy to improve outcomes in patients with poor outcomes^8,35^. Similarly, de-escalation strategies can be considered in patients with an expected better prognosis in order to avoid treatment-related toxicities. Recent studies exploring treatment de-escalation with the replacement of concurrent cisplatin with cetuximab in HPV-positive oropharyngeal cancer resulted in negative results^36,37^. These results explored a generalized approach using HPV as a marker of de-intensification of chemotherapy but turned out to be ineffective. The use of a QUS-based radiomics model can help in predicting clinical radiosensitivity aiding in better risk-stratification on an individual patient basis and guiding towards precision medicine.

The current analysis involved a relatively small number of patients. The study here is currently being continued, and with further expansion, it is expected to lead to the generation of more robust and reproducible imaging biomarker models. Thus study included binary endpoints at two different times due to a small number of samples. With the inclusion of more patients, advanced classification algorithms like deep learning strategies and more robust validation strategies can be undertaken. In addition, given the technical challenges of imaging primary head and neck tumours (deep location, tissue interfaces), in the patients here, the neck node was scanned. Given the high correlation of response of both the primary tumour and index neck node following treatment, using nodal imaging features alone suggests the clinical utility of the QUS-radiomics methods evaluated in the work here.

## Methods and materials

### Patient selection

**Figure 5:**
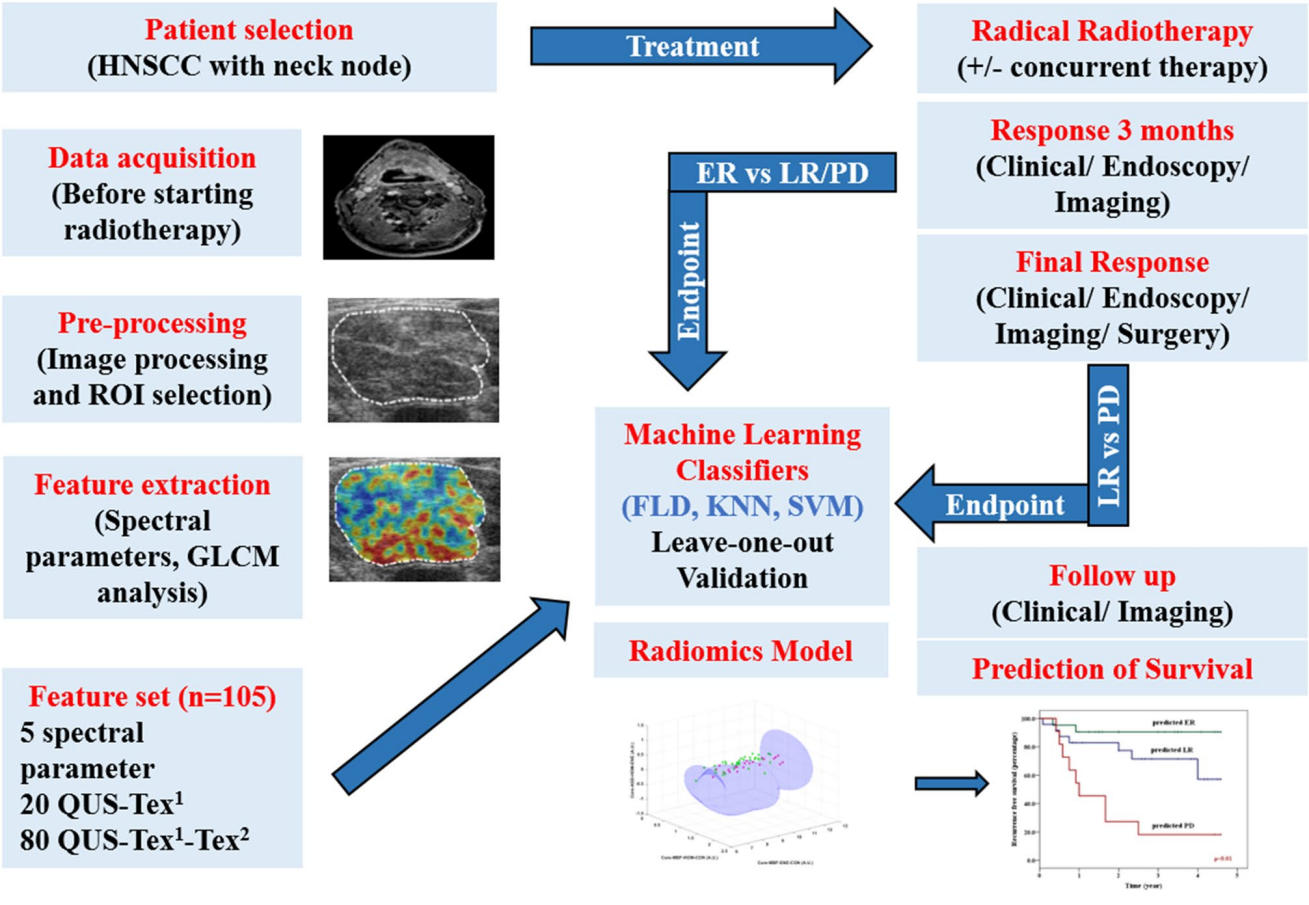
Flowchart showing the study methodology.

The prospective study was conducted based on good clinical practice according to the declarations of Helsinki and institutional research ethics board approval (Sunnybrook Health Sciences Center, Canada) and registered with clinicaltrials.gov (identifier NCT03908684, registered on 09/04/2019). Patients with a diagnosis of biopsy-proven HNSCC with metastatic LN, the latter amenable to ultrasound imaging from a primary disease involving oropharynx, hypopharynx, larynx, or carcinoma of an unknown primary (CUP) treated with radical RT were eligible for this study. Patients with poor performance status (Eastern Co-operative Oncology Group > 1), prior treatment for HNSCC, previous history of RT to the head and neck region, severe medical or psychiatric comorbidities with a life expectancy of < 6 months or unreliable for follow up were considered as exclusion criteria. Informed consent was obtained from all patients accrued in this study. Patients with nasopharyngeal cancer (NPC), CUP suspected from NPC (Epstein-Barr virus positivity or histological suspicion), oral cavity primary or no follow up after treatment completion for response assessment were excluded from the current analysis. The study methodology has been represented in Fig. 5.

### Treatment protocols and response evaluation

Study participation had no influence on patient treatment or follow up. All patients were treated using a uniform radiation dose regimen of 70 Gy/33–35 fractions over 6–7 weeks to the high-risk volume using IMRT and IGRT techniques according to standard institutional practice. The use of concurrent systemic therapy was at the discretion of the responsible medical oncologist. The first response to treatment was evaluated at approximately 3 months following completion of RT with clinical examination, endoscopy, and morphological imaging with CT/MRI supplemented with PET-CT. Patients with complete resolution of the primary tumour and index lymph node < 1 cm at the end of 3 months were considered as “Early Responders” (ER). Patients with a residual nodal size of < 1 cm with strong radiological suspicion of residual disease or histological confirmation were excluded from the ER category. Patients with partial response or non-responders at 3 months were further followed up with clinical examinations, additional imaging, histopathological examination, or surgical intervention. Patients who experienced complete resolution of disease beyond 3 months were labelled as “Late Responders” (LR). Other patients were labelled as “persistent or progressive disease” (PD) involving the primary or index LN. Further follow up was done every 3–6 months in the initial 2 years and thereafter every 6–12 months as indicated. Study accrual was carried out between January 2015 and October 2019, with the final analysis done in June 2020.

### Image acquisition

Ultrasound RF data were collected from the index LN most amenable to imaging using a Sonix RP ultrasound imaging system (Ultrasonix, Vancouver, Canada) or an equivalent OEM system from Elekta Ltd (Montreal, Canada) before starting RT. Imaging was performed by a research sonographer with experience in head & neck ultrasound imaging. The system used a linear array transducer (Elekta: 4DL14-5/39, Sonix RP: L14-5/60) with a center frequency of 6.5 MHz with a bandwidth of 3–8 MHz. A 40 MHz sampling frequency was used for digitization. Data were acquired along 512 scan lines, spanning a 6 cm lateral field-of-view and the focal depth of 1.75 cm (Sonix RP) and 2.5 cm (Elekta). Earlier work has indicated that with appropriate data normalization using standardized techniques, QUS features are consistent between different clinical ultrasound devices^38^. The index LN was manually contoured on US B-mode images and designated as the region of interest (ROI). For each patient, typically 3–5 slices were selected from the entire ROI at regular intervals. Given the technical challenges in volumetric acquisition and analysis of conventional probe-based ultrasound, the slices were selected to represent different areas of the lymph node. Quantitative ultrasound spectroscopic analysis, associated texture analysis, and texture-derivate analyses were performed on the selected regions from the target LN as described below.

### Quantitative ultrasound spectral features

Spectral analysis was performed using the RF data associated with the segmented area of the index LN. A sliding window analysis was used with a 2-mm by 2-mm kernel to create parametric images for each QUS spectral parameter. A 94% overlap was used between adjacent sliding windows in both axial and lateral directions. Prior to spectral analysis, a Hanning gating function was applied on individual RF scan lines within the window. The power spectrum was generated using a Fast Fourier Transform (FFT) method. An average power spectrum was obtained from RF signals within the window. The power spectrum was normalized using a reference phantom technique using 5–30 µm glass beads embedded in a homogeneous medium of oil droplets embedded in gelatin^39,40^. For the phantom, the measured attenuation coefficient and speed of sound were 0.8 dB/cm/MHz and 1540 m/s, respectively (the University of Wisconsin, Department of Medical Physics, Madison, WI, USA). Attenuation compensation was applied to account for the attenuation from the intervening tissue layers (intervening tissue and tumour) considering 1 dB/cm/MHz for the overlying breast tissues^39,41^. The attenuation coefficient estimate (ACE) for the tumour was determined using a spectral difference method. This considered the rate of change in the log spectral power magnitude for the frequency bandwidth with depth (over the tumour region) relative to the reference phantom^39,40^. Five spectral parameters were determined from the power spectrum-mid-band fit (MBF), spectral slope (SS), spectral intercept (SI), average scatterer diameter (ASD), and average acoustic concentration (AAC). Further details and the biological correlations of individual spectral parameters have been described in previous publications^33,42^. The values of the different spectral features were obtained from the individual windows, and the average weighted values from all the slices were used as first-order imaging features.

### Texture parameters

Colour-coded quantitative ultrasound-based parametric maps were generated based on the values of individual spectral parameters from each of the corresponding windows. A grey level co-occurrence matrix (GLCM) method was used to determine texture features to quantify intra-tumoural heterogeneity^43^. The GLCM method analyses the spatial relationship between neighbouring pixels at different angular directions. The grey level intensities of each of the parametric images were linearly scaled into 16 discrete values. The GLCM matrices were created from each QUS-parametric map at inter-pixel distances: 1, 2, 3, 4, 5 pixels and at four angular directions: 0°, 45°, 90°, and 135°. Four textural features of contrast (CON), correlation (COR), energy (ENE), and homogeneity (HOM) were extracted and subsequently averaged over distances and angular directions to generate second-order imaging features (QUS-Tex^1^). Therefore, 5 spectral features led to the generation of 20 QUS-Tex^1^ features.

The third-order imaging features were texture-derivatives (QUS-Tex^1^-Tex^2^). A previous study involving breast cancer had demonstrated an improvement in the classifier performances with the inclusion of higher-order imaging features in the form of texture derivatives^44^. Texture-derivate analysis was done by creating intermediate texture-encoded maps using sliding window analysis with a 15-pixel by 15-pixel window with each pixel in these maps representing quantification of local textures within the concerned window. A second pass GLCM analysis was performed on the texture maps, resulting in 80 QUS-Tex^1^-Tex^2^ features.

The weighted averaged measures of the features were used for building models for predicting response. A total set of 105 QUS-radiomic features (5 spectral, 20 QUS-Tex^1^, 80 QUS-Tex^1^-Tex^2^) were acquired before starting RT. Imaging was obtained nominally within 24 h in advance of the start of treatment, although an interval of 7 days was allowed according to the study protocol.

### Statistical analysis

Binary endpoints were used for constructing the radiomics model due to a relatively smaller number of patients in our study, and multiple classifier outputs will need a larger sample size for robust classification. The first endpoint was the response at 3 months following completion of RT-complete responders vs. partial/non-responders (i.e. ER vs LR + PD). The other classification endpoint was LR vs PD.

The distribution of categorical variables (clinical features) between the three response groups was studied using the Pearson chi-square test and Fisher’s exact test as appropriate. For the feature values, a Shapiro–Wilk test was performed to test the normality of distribution. For comparison between the binary response groups, an unpaired *t-*test was used for normally distributed data, while a Mann–Whitney U-Test was used for non-parametric data. The survival analysis was performed using the Kaplan–Meier product-limit method, with the date of starting RT considered as baseline. A comparison of the different factors on survival was conducted using a log-rank test. A p-value of < 0.05 was used as a threshold of statistical significance.

### Machine learning classifiers

Three standard computational algorithms were used for the development of a radiomics model: Fischer’s linear discriminant analysis (FLD), *k-*nearest neighbours (KNN), and support vector machines-radial basis function (SVM-RBF). The FLD is a linear classification algorithm exploring multi-dimensional feature space that maximizes the ratio between-class to within-class variance. The KNN is an instance-based classification algorithm studying class association of a test point in the feature space. It is based on the spatial distribution of most of the points neighbouring the test point and the distance between those points to the test point. The classifier was tested using *k*-values of 1 to 5 nearest neighbours to find the one leading to the most optimal classification. The SVM-RBF algorithm used a nonlinear classification that maximizes the margin between the two specified classes. The input data was mapped into a higher-dimensional space using kernel functions where the data are supposed to have better distribution, which then applied a hyperplane optimally separating the two classes in this higher-dimensional feature space. A Gaussian radial basis function (RBF) as the kernel function was used in our study. The model parameters (soft margin parameter C and the free parameter γ) were optimized using a grid search method.

A forward sequential-feature-selection (SFS) technique was used for classification using predetermined endpoints. To avoid the overfitting of the predictive model, the number of features for the final classification was limited to 3 for ER vs. LR/PD and 2 features for LR vs. PD^45^. The classifier performance was trained based on F1-Score (the harmonic average of precision and sensitivity). A leave-one-out (LOO) cross-validation technique was used for validation and testing the efficacy of the classifier performances, which involves training the classification model with all observations except one which was used to test the developed model. Receiver operating characteristics (ROC) analysis was performed utilizing sensitivity, specificity, accuracy, and area under curve (AUC). The segmentation, feature extraction, texture analysis, and machine learning classification were done using MATLAB 2019b (MathWorks Inc., USA). Survival analysis was performed using IBM SPSS version 21 (IBM corporation).

## Conclusions

The use of a pretreatment texture-derivative based QUS-radiomics model was able to predict the final response to radiation with excellent accuracy (more than 90%). Clinical radiosensitivity had a strong influence on the survival outcomes, and a support vector machine classifier could accurately identify patients at higher risk of disease recurrence based on QUS-predictive factors.

## Supplementary Information

Supplementary Legend.

Supplementary Figure S1.

## Data Availability

Data will be shared upon request to the corresponding author and the institutional ethics committee according to the policy of Sunnybrook Health Sciences Centre, Toronto.
