# Supplementary material for: Assessment of clinical radiosensitivity in patients with head-neck squamous cell carcinoma from pre-treatment quantitative ultrasound radiomics
Source: Sci Rep. 2021 Mar 17;11:6117. doi: 10.1038/s41598-021-85221-6 (PMC7969626; doi:10.1038/s41598-021-85221-6)
Supplement: Supplementary file 1 — Supplementary Legend. [file 41598_2021_85221_MOESM1_ESM.docx]

**Supplementary figure 1:** The scatter plot representing all the patients for individual 105 features between the complete responder (CR) and partial responder (PR)/ non-responder (NR) at 3 months following radiotherapy and between the late responder (LR) and persistent disease/ progressive disease (PD) (final response for patients with partial or no response at 3 months).
