# Supplementary material for: Assessment of clinical radiosensitivity in patients with head-neck squamous cell carcinoma from pre-treatment quantitative ultrasound radiomics
Source: Sci Rep. 2021 Mar 17;11:6117. doi: 10.1038/s41598-021-85221-6 (PMC7969626; doi:10.1038/s41598-021-85221-6)

Title: Assessment of Clinical Radiosensitivity in Patients with Head-Neck Squamous Cell Carcinoma from Pretreatment Quantitative Ultrasound Radiomics

Authors:
Laurentiusoscar Osapoetra[1-3*], Archya Dasgupta[1-3*], Daniel Di Cenzo[3], Kashuf Fatima[3], Karina Quiaoit[3], Murtuza Saifuddin[3], Irene Karam[1-2], Ian Poon[1-2], Zain Husain[1-2], William T. Tran[1,2,4], Lakshmanan Sannachi[3], Gregory J. Czarnota[1-3,5]
Affiliations:

1. Department of Radiation Oncology, Sunnybrook Health Sciences Centre, Toronto, Canada
2. Department of Radiation Oncology, University of Toronto, Toronto, Canada
3. Physical Sciences, Sunnybrook Research Institute, Toronto, Canada
4. Evaluative Clinical Sciences, Sunnybrook Research Institute, Toronto, Canada Department of Medical Oncology, Department of Medicine, Sunnybrook Health Sciences Centre, Toronto, Canada
5. Department of Medical Biophysics, University of Toronto, Toronto, Canada

*L.O and A.D made equal contributions to this study.

Corresponding Author
Gregory J. Czarnota, Ph.D., MD
Sunnybrook Health Sciences Centre, T2 167, 2075 Bayview Avenue,
Toronto, Ontario, Canada, M4N3M5
Tel: 416 480 6128, Email: gregory.czarnota@sunnybrook.ca

Supplementary Figure 1

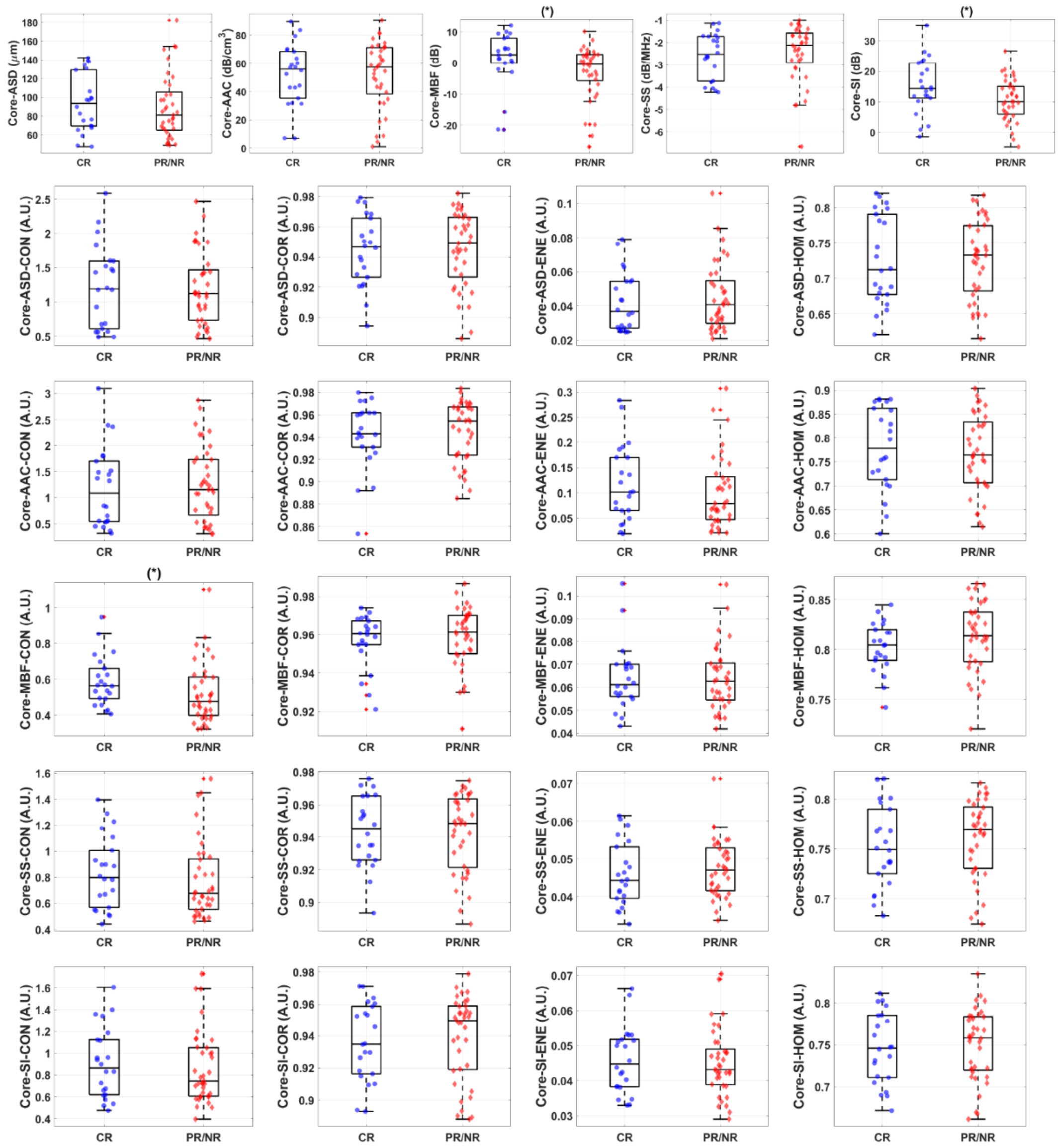

Supplementary Figure 1

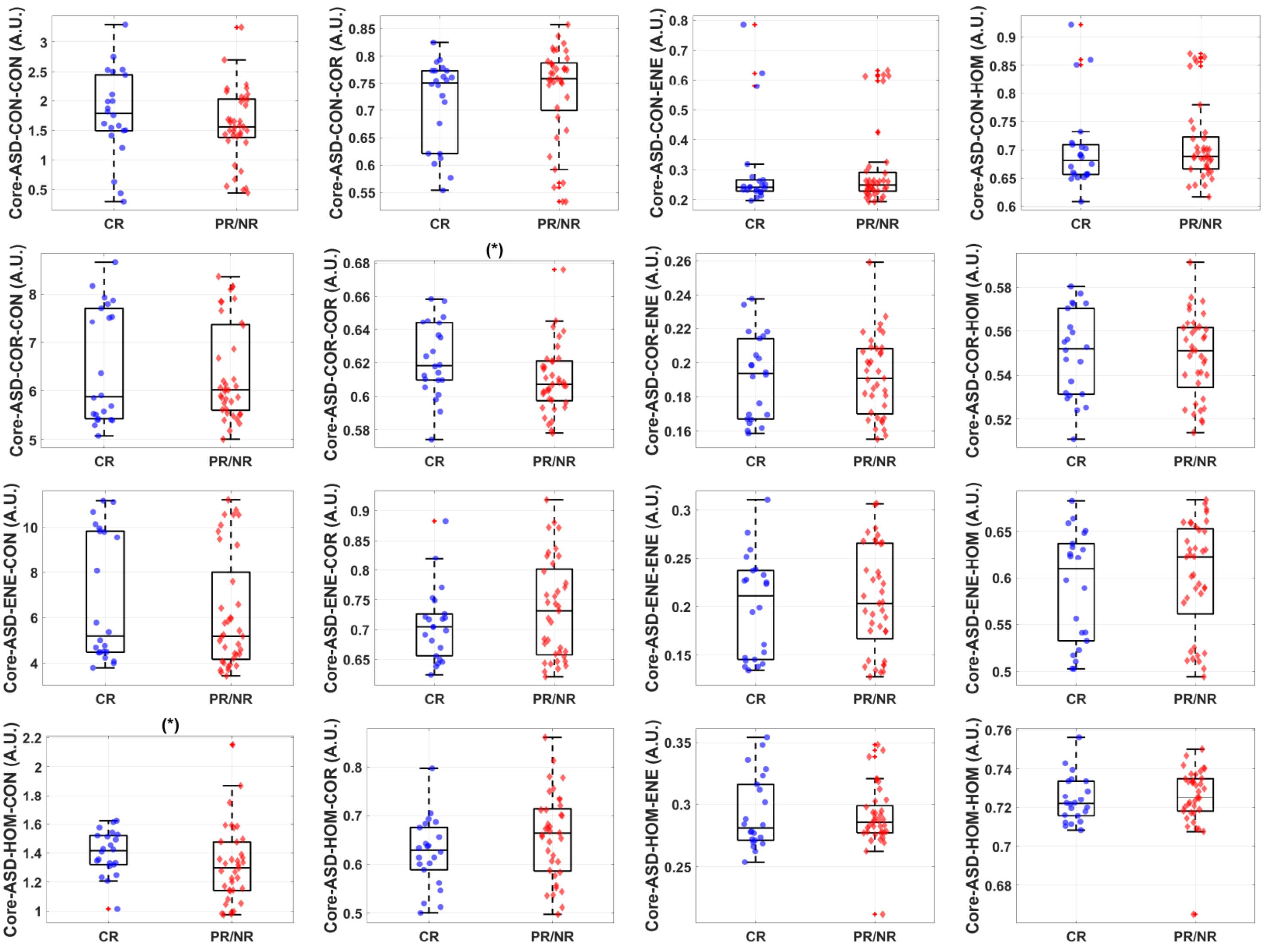

Supplementary Figure 1

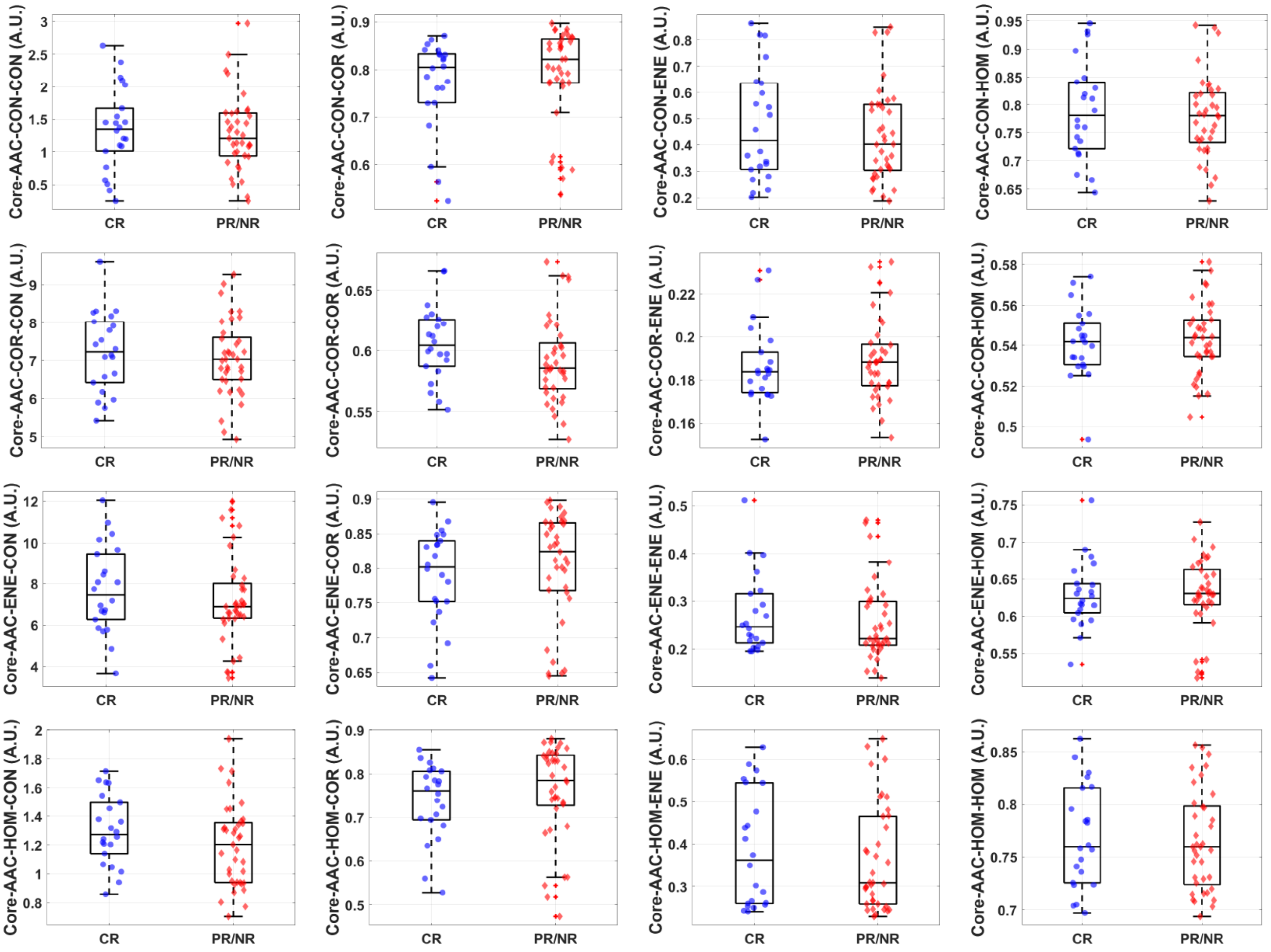

Supplementary Figure 1

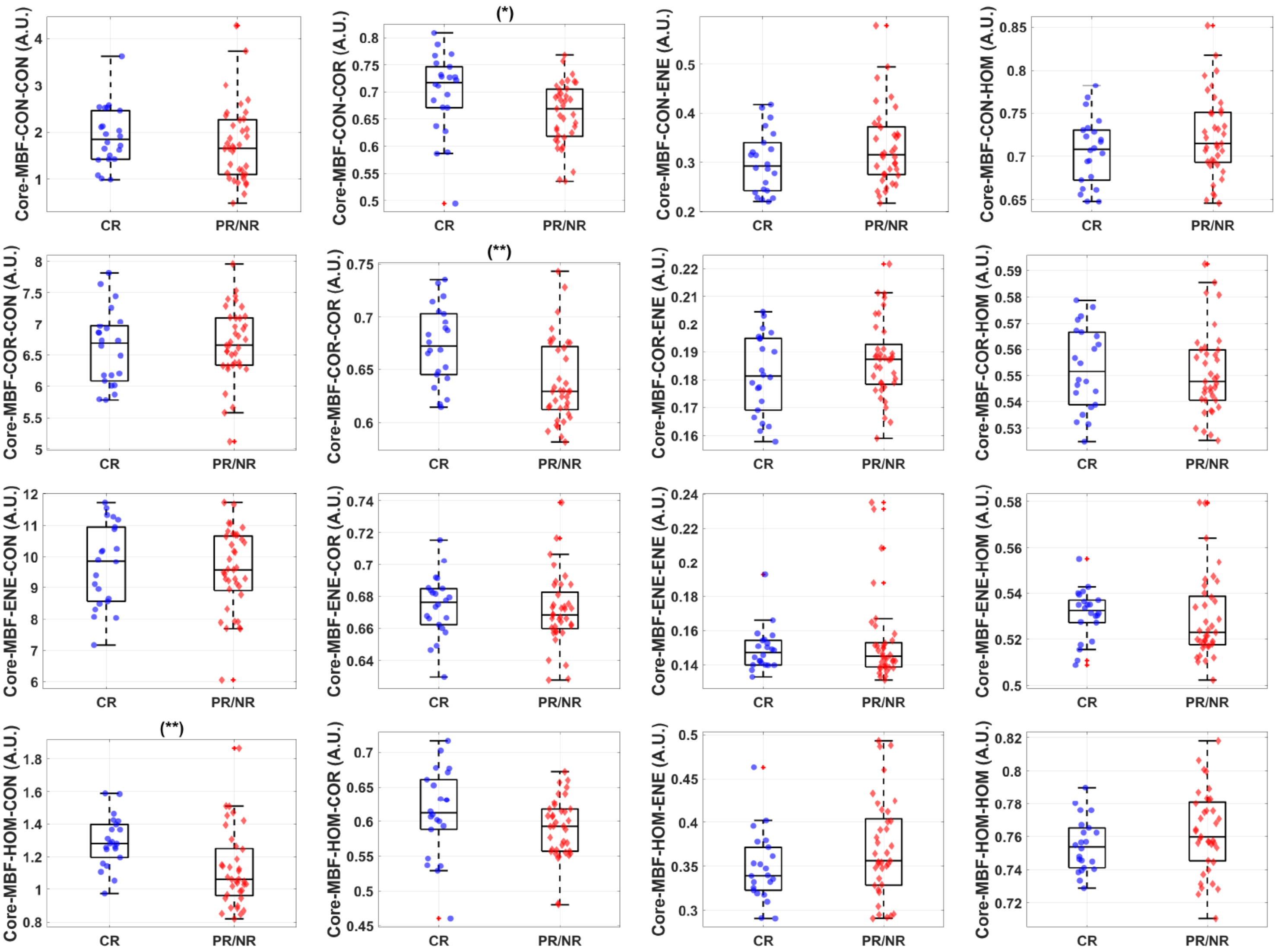

Supplementary Figure 1

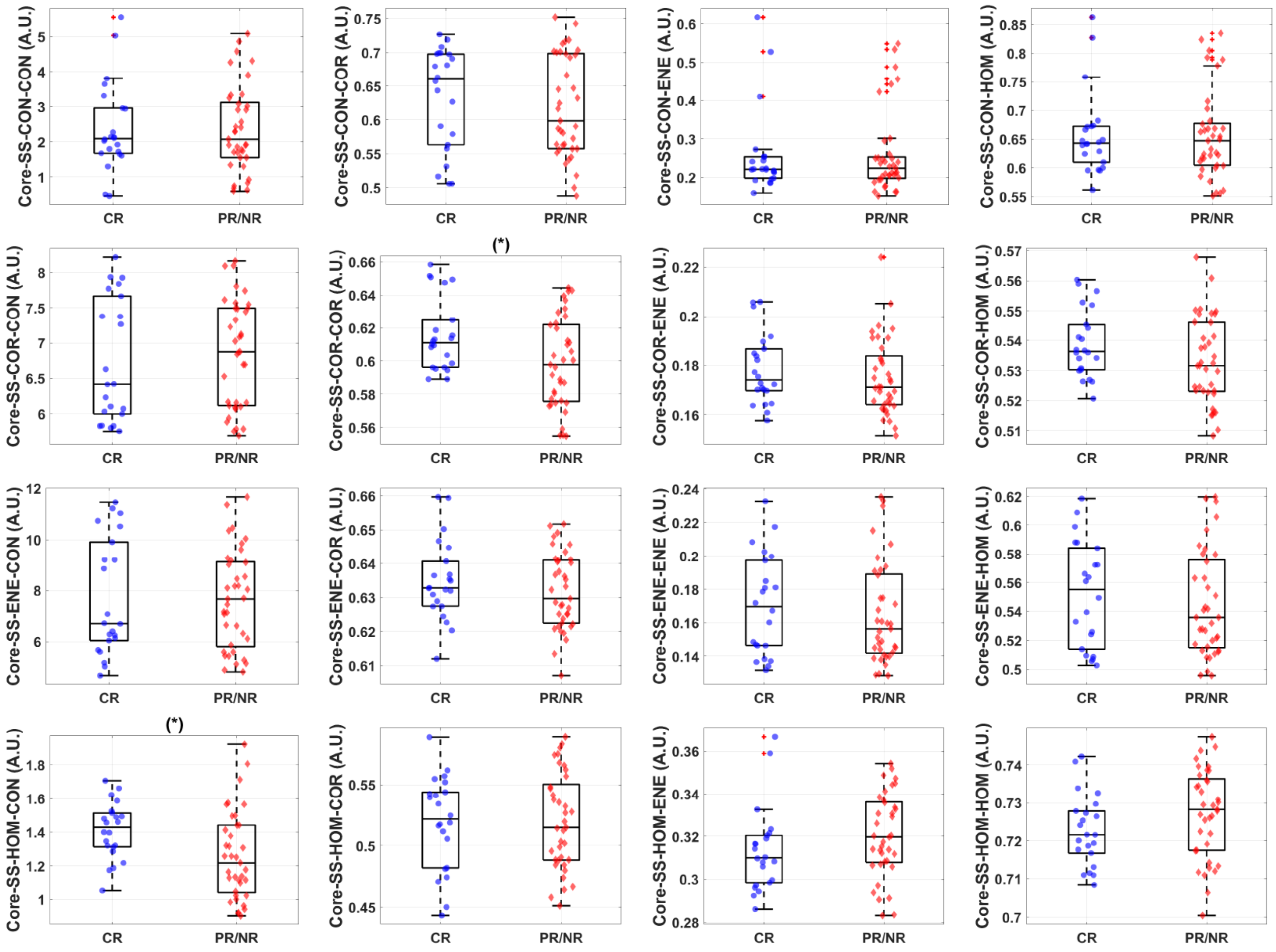

Supplementary Figure 1

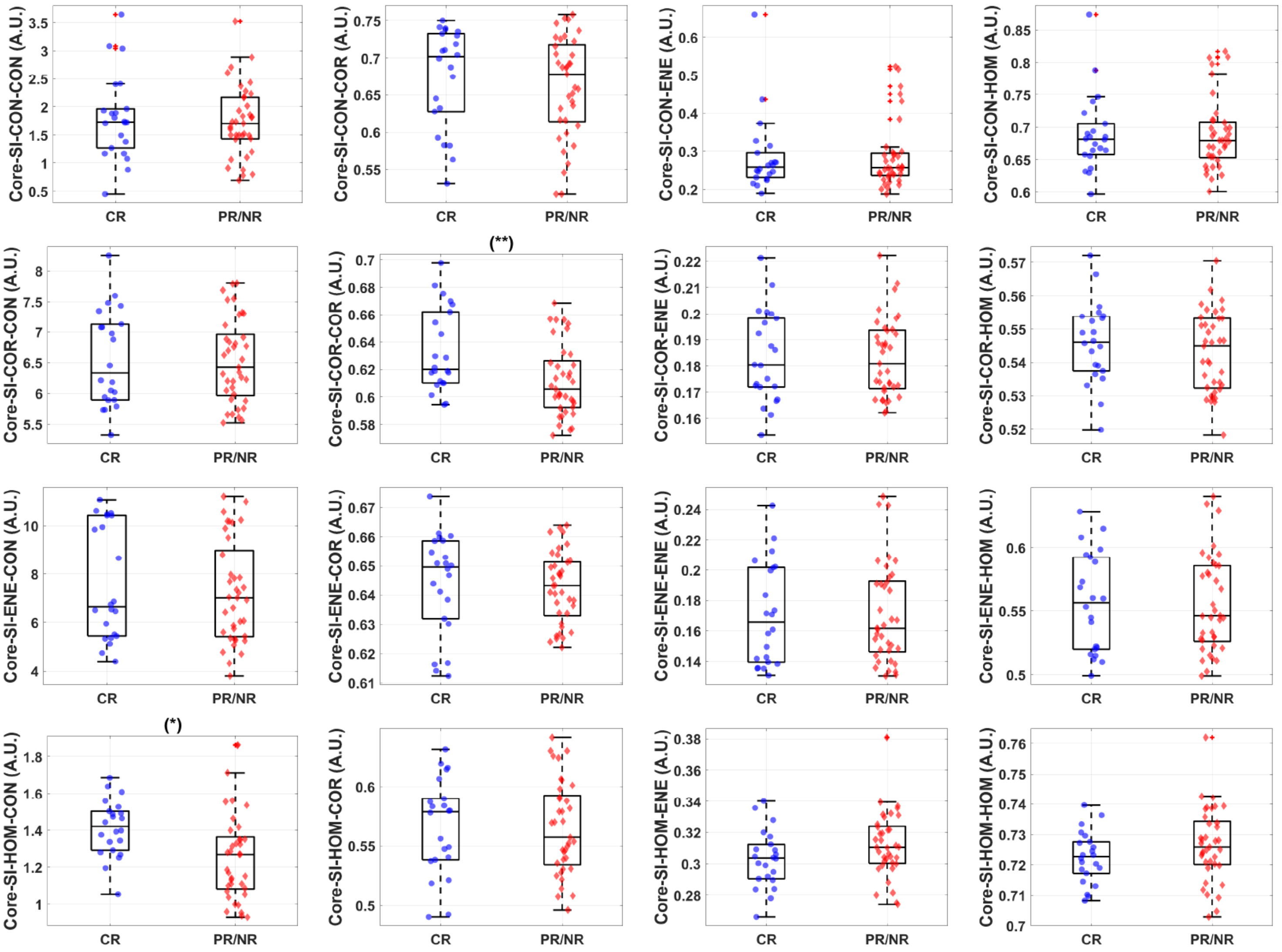

Supplementary Figure 1

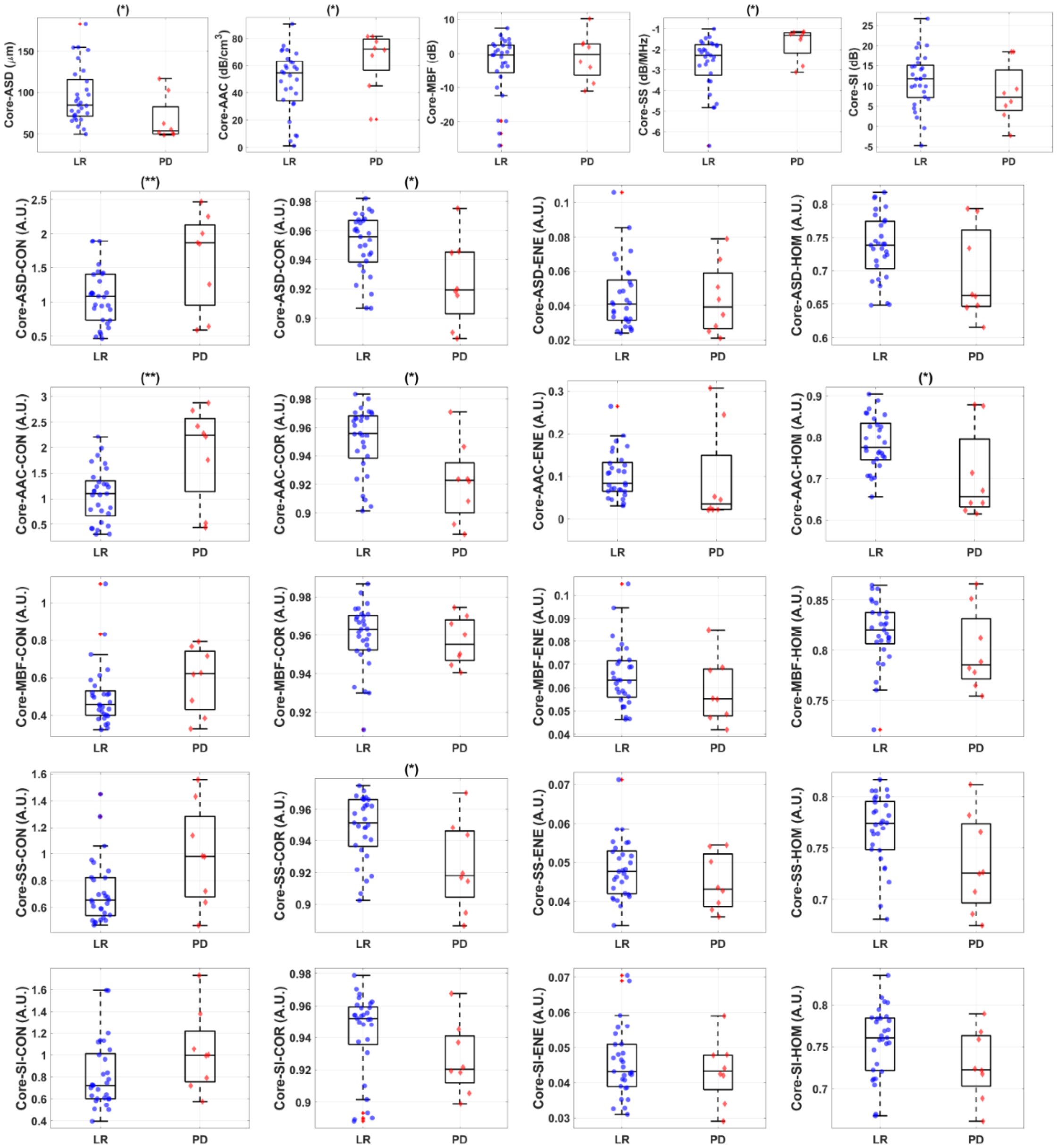

Supplementary Figure 1

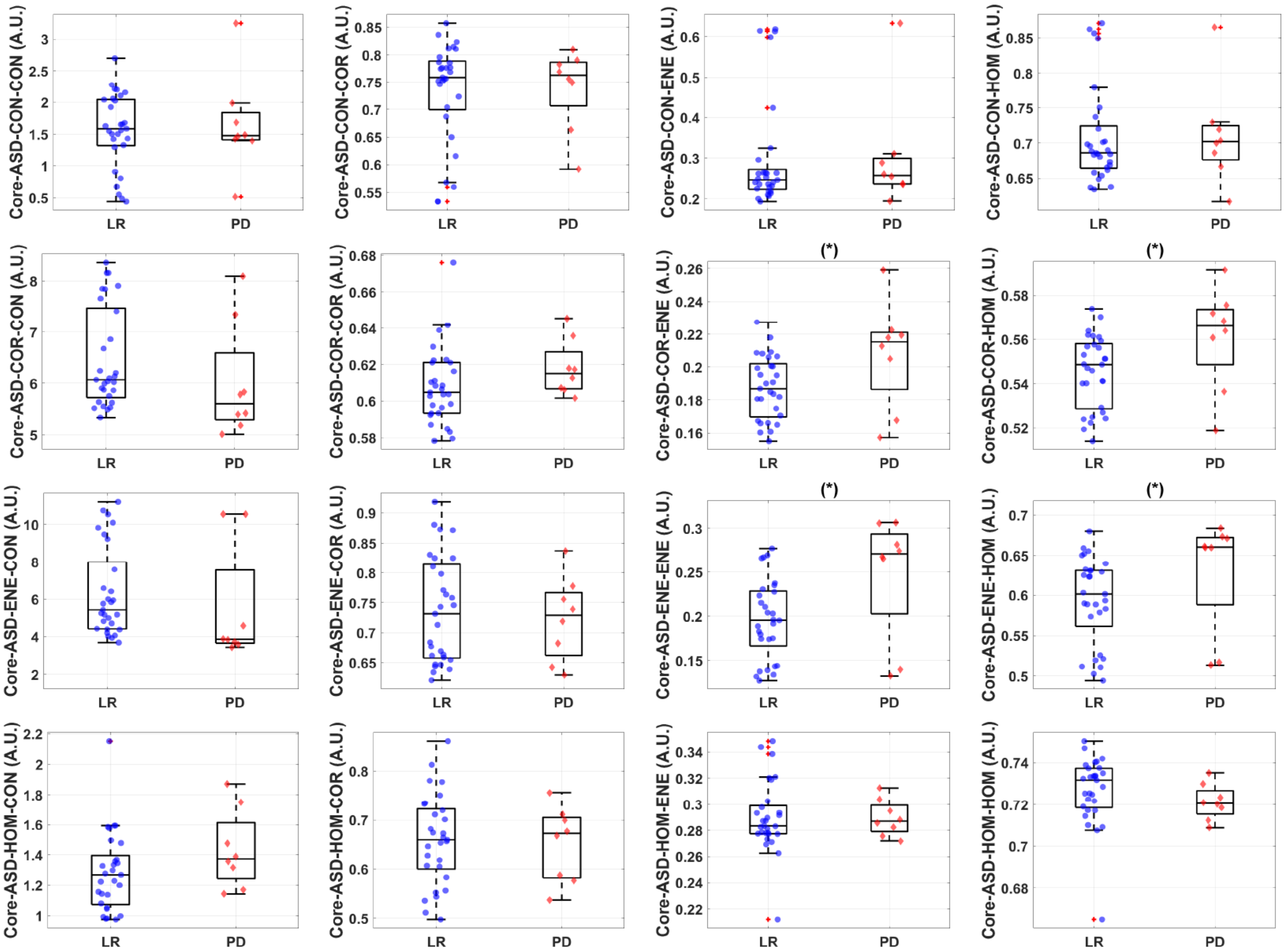

Supplementary Figure 1

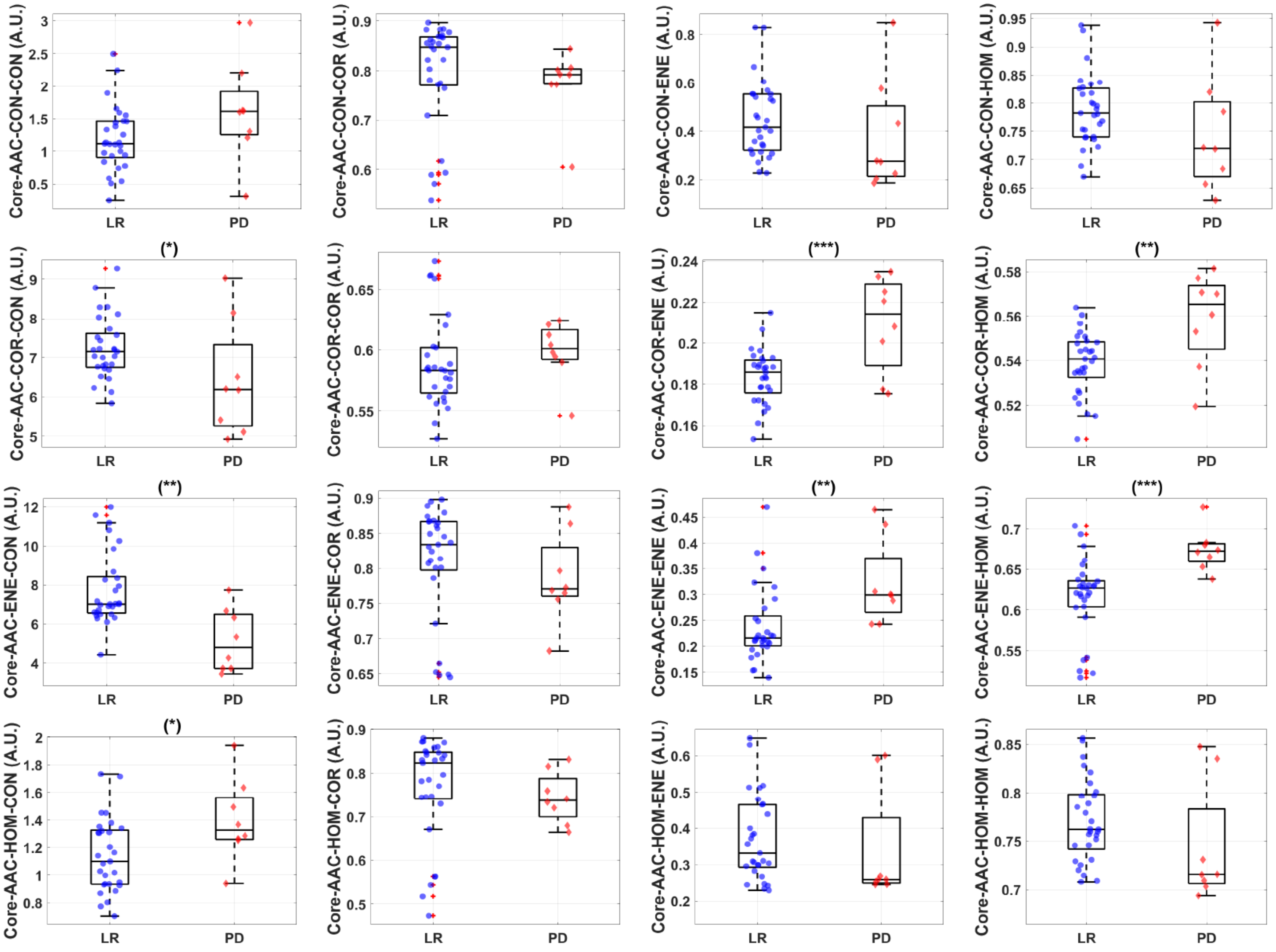

Supplementary Figure 1

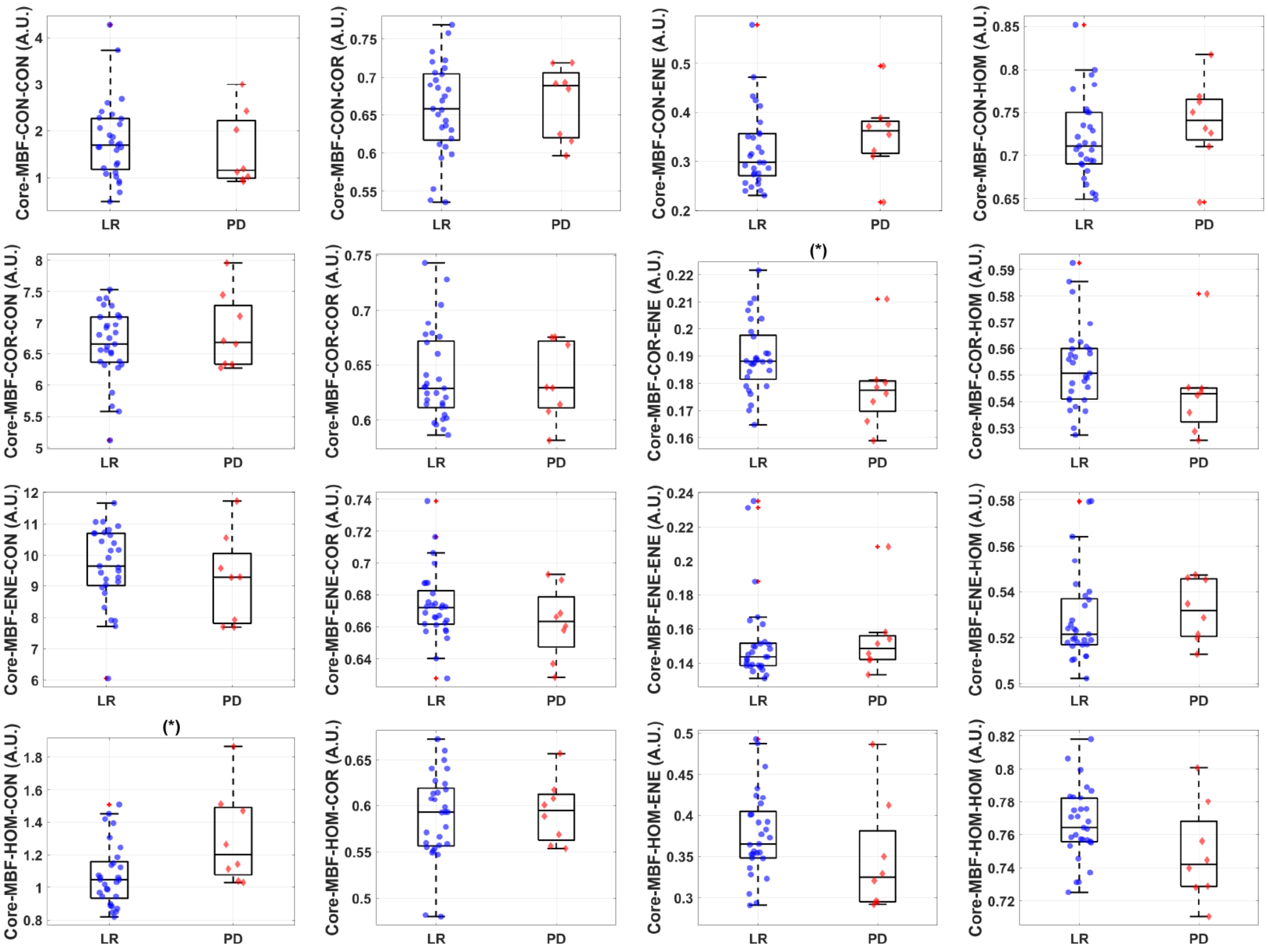

Supplementary Figure 1

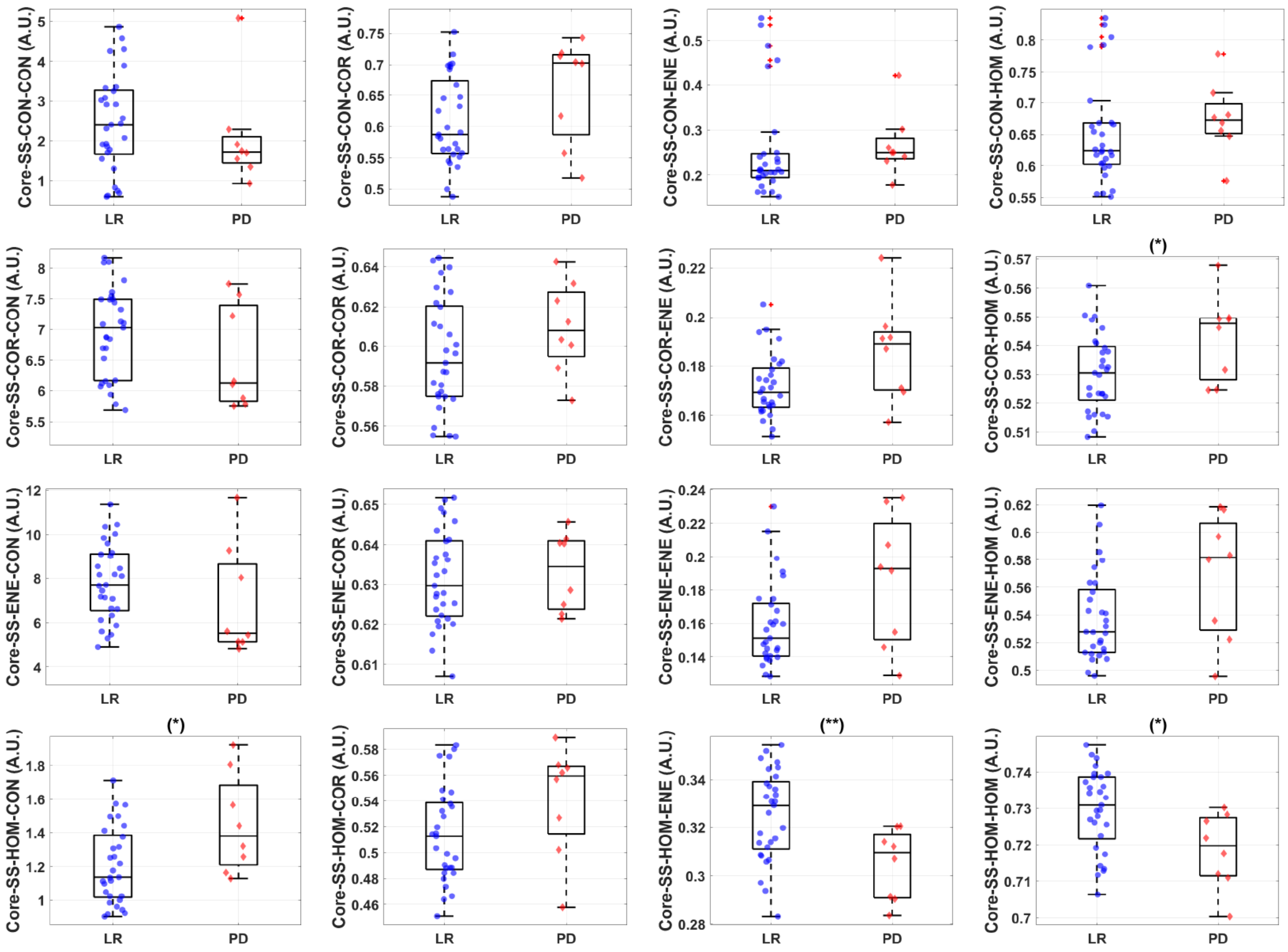

Supplementary Figure 1

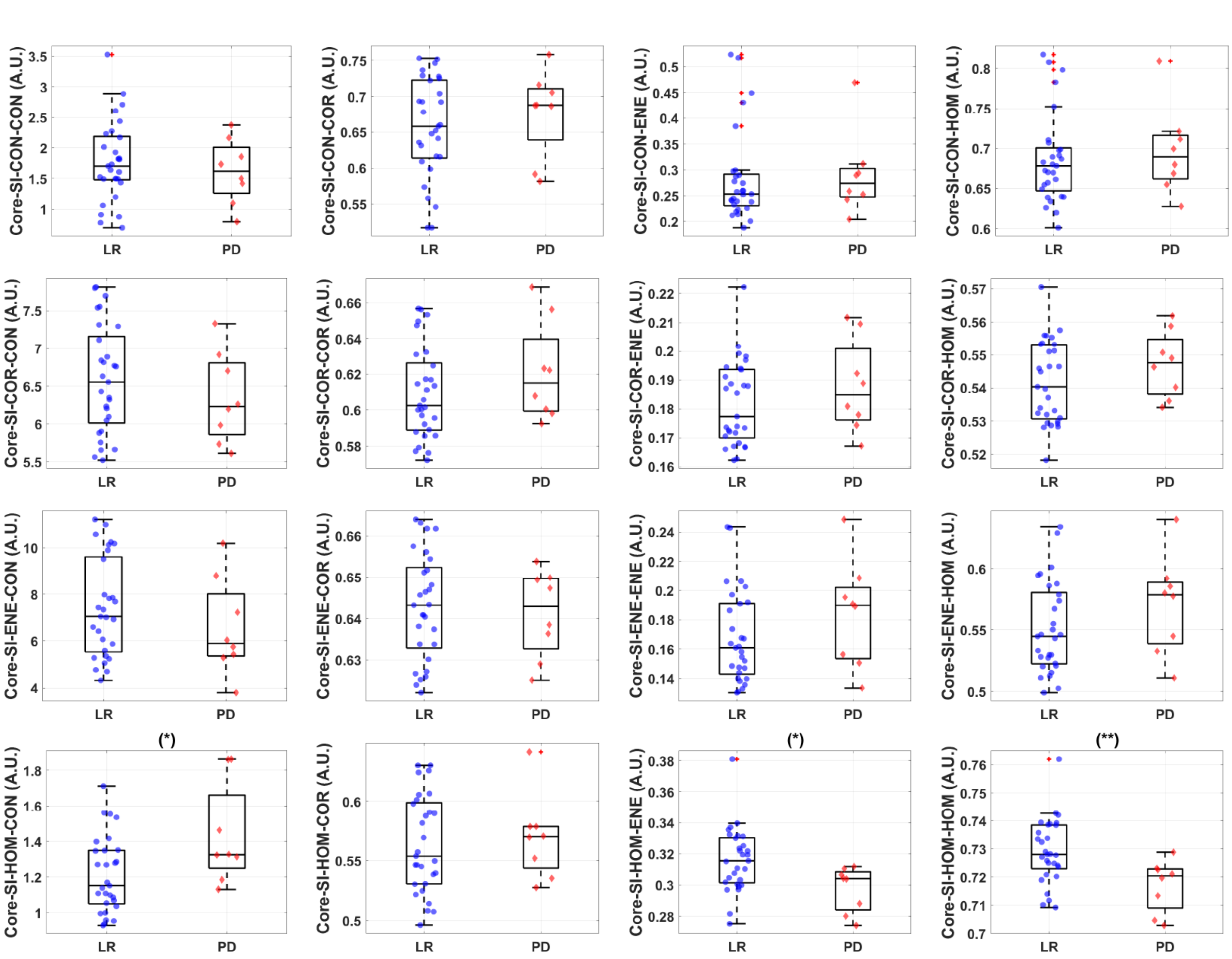

Supplement: Supplementary file 2 — Supplementary Figure S1. [file 41598_2021_85221_MOESM2_ESM.pdf]
